# Assessing Consumer Health Vocabulary Familiarity: An Exploratory Study

**DOI:** 10.2196/jmir.9.1.e5

**Published:** 2007-03-14

**Authors:** Alla Keselman, Tony Tse, Jon Crowell, Allen Browne, Long Ngo, Qing Zeng

**Affiliations:** ^3^Decision Science GroupBrigham and Women’s HospitalHarvard Medical SchoolBostonMAUSA; ^2^AquilentIncLaurelMDUSA; ^1^National Library of MedicineNational Institute of HealthBethesdaMDUSA

**Keywords:** Consumer health vocabulary, Patients, Vocabulary, Consumer health informatics, Health education, Readability, Comprehension, Health, Evaluation studies

## Abstract

**Background:**

Accurate assessment of the difficulty of consumer health texts is a prerequisite for improving readability. General purpose readability formulas based primarily on word length are not well suited for the health domain, where short technical terms may be unfamiliar to consumers. To address this need, we previously developed a regression model for predicting “average familiarity” with consumer health vocabulary (CHV) terms.

**Objective:**

The primary goal was to evaluate the ability of the CHV term familiarity model to predict (1) surface-level familiarity of health-related terms and (2) understanding of the underlying meaning (concept familiarity) among actual consumers. Secondary goals involved exploring the effect of demographic factors (eg, health literacy) on surface-level and concept-level familiarity and describing the relationship between the two levels of familiarity.

**Methods:**

Survey instruments for assessing surface-level familiarity (45 items) and concept-level familiarity (15 items) were developed. All participants also completed a demographic survey and a standardized health literacy assessment, S-TOFHLA.

**Results:**

Based on surveys completed by 52 consumers, linear regression suggests that predicted CHV term familiarity is a statistically significantly predictor (*P* < .001) of participants’ surface-level and concept-level familiarity performance. Health literacy was a statistically significant predictor of surface-level familiarity scores (*P* < .001); its effect on concept-level familiarity scores warrants further investigation (*P* = 0.06). Educational level was not a significant predictor of either type of familiarity. Participant scores indicated that conceptualization lagged behind recognition, especially for terms predicted as “likely to be familiar” (*P* = .006).

**Conclusions:**

This exploratory study suggests that the CHV term familiarity model is predictive of consumer recognition and understanding of terms in the health domain. Potential uses of such a model include readability formulas tailored to the consumer health domain and tools to “translate” professional medical documents into text that is more accessible to consumers. The study also highlights the usefulness of distinguishing between surface-level term familiarity and deeper concept understanding and presents one method for assessing familiarity at each level.

## Introduction

Improving the readability of online consumer health materials is an important area of eHealth research. Studies indicate that health information on the Web is beyond the reading ability of average consumers [1,2]. Research on general literacy suggests that readability decreases as the number of “difficult” words, those unfamiliar to the average reader, increases. Since familiarity correlates with education and literacy levels, “easy” terms are those that are familiar to many individuals who have lower reading skills. For example, the Dale-Chall readability formula incorporates a list of 3000 words and phrases (expressions) familiar to 80% of fourth-grade students in the United States [3]. However, because obtaining a comprehensive, empirically derived list of familiar words is difficult, many other existing readability formulas use average number of syllables per word as a surrogate for word difficulty.

Many researchers point to the need to reduce the gap between health literacy of the readers and the readability of consumer health materials [4]. As guidelines call for using simple common words, adhering to them requires predicting consumer familiarity with various health-related words. Currently, the only available methods are general purpose readability formulas developed by K-12 researchers. However, using such readability formulas to predict readers’ ability to comprehend health texts has been criticized by the health literacy community. As McCray observes, “counting words and syllables and consulting a grade-level word list are most likely not sufficient to determine how readable a text is” [5]. Reliance on word length is particularly ill suited for the health domain, where short technical terms are likely to be unfamiliar to consumers (eg, apnea). The logic of graded word lists simplifies the phenomenon of word knowledge by implying that it is binary in nature and suggests that a reader is either unfamiliar or familiar with a particular word, with the switch between not knowing and knowing occurring at a single point in time. However, consumer health term familiarity is a more nuanced phenomenon involving partial knowledge [6], and increased exposure likely results in increased familiarity.

Recognizing the limitations of these previous approaches, we set out to explore alternative measures that account for “average” familiarity with health terms among members of a convenience sample of consumers. The ability to recognize terms is important because readers need to associate health terms with their corresponding concepts in order to extract useful information from text. Thus, we decompose health vocabulary knowledge into two parts: (1) surface-level term familiarity, or recognition of the lexical form, and (2) concept-level term familiarity, or understanding of the underlying concept. In cognitive science, a concept can be viewed as a set of slots that can be filled with characteristics describing a class of objects or events [7]. For instance, a “disease” concept may be characterized by attributes such as cause, severity, duration, and pathophysiology (among others). The completeness and accuracy of conceptual knowledge exists on a continuum, dictated by context. Thus, a healthy individual with a family history of diabetes and a diabetic patient may each benefit from explanations focusing on different aspects of diabetes (eg, prevention versus treatment). Yet, historically, readability studies do not distinguish between surface-level lexical forms (commonly referred to as “terms”) and concepts and, therefore, do not separately assess familiarity at each “level.”

 We had previously developed a support vector machine regression model for predicting “familiarity likelihood scores” of consumer health vocabulary (CHV) terms using the empirical data from user studies evaluating “consumer-friendly display” names for medical concepts [8] as training data and the term frequency counts from health text corpora as features [9]. The model evaluated by this current study was an improved version of the initial model published in 2005 [9]: actual familiarity data were collected from 41 subjects for training, and term and word frequencies in three different corpora were used as features, including (1) Reuters news reports (health and non-health articles), (2) queries to a health search engine (MedlinePlus), and (3) queries to a general search engine (MetaCrawler). This algorithm assigns each consumer health term with a predictive score ranging from 0 to 1.0, representing the likelihood that a term is familiar to the average consumer. Terms are classified into three familiarity categories based on their scores: “likely” (> 0.8), “somewhat likely” (0.8-0.5), and “not likely” to be familiar (scores < 0.5).

The primary goal of the research reported in this paper was to develop and apply a simple methodology for validating the CHV familiarity predictive model against actual empirically derived familiarity with various health terms among health consumers. The validation is distinct and independent from the empirical data used in deriving the model. Both surface-level (ie, recognition) and concept-level familiarity (ie, understanding of the underlying meaning) data were collected from participants. Surface-level familiarity was investigated because it corresponds with existing conventional approaches to assessing health vocabulary knowledge. The goal of concept-level familiarity assessment was to explore the potential of this novel approach and to characterize the relationship between the two familiarity levels. Finally, we sought to describe the effect of demographic factors (including health literacy and education level) on actual consumers’ scores. The following three hypotheses addressed the goals of the study:

1. Predicted familiarity likelihood level will have a significant effect on consumer surface-level term familiarity and consumer understanding of the underlying concept.
2. Demographic factors, including but not limited to health and education level, will have a significant effect on both types of familiarity scores.
3. Consumers’ surface-level familiarity with terms will be greater than their understanding of the underlying concepts.

## Methods

### Participants

Consumers (n = 52) were recruited from Brigham and Women’s Hospital. Health literacy, assessed with Short Test of Functional Health Literacy in Adults (S-TOFHLA) [10], ranged in score from 22 to 36 (mean = 33.04; SD = 3.83). Based on these scores, 50 participants had adequate health literacy skills (scores from 23-36 out of 36), while two had marginal skills (scores from 17-22).

 Other demographic variables were self-reported using a brief questionnaire (Table 1). There were 8 non-native English speakers, with number of years speaking English ranging from 6 to 40 (median = 12 years). The level of English proficiency was not assessed, as the complexity of the relationship between primary and secondary language health literacy is beyond the scope of this study. Of the 8 non-native English participants, 7 achieved S-TOFHLA scores in the high literacy range, and the remaining participant, in the moderate literacy range (self-reported as speaking English for 40 years).

**Table 1:** Demographic characteristics of the participants (n = 52)

**Demographic Variable**	**Number**
**Gender**	
Male	16
Female	36
**English proficiency**	
Native speakers	44
Non-native speakers	8
**Highest education level**	
Below high school	2
High school	9
Some college	20
College	13
Graduate school	8
**Age**	
18-25	5
26-39	13
40-59	25
≥ 60	9
**Race**	
White	25
Black	13
Hispanic	8
Other	6
**Health literacy level (STOFHLA scores)**	
high health literacy (23-36)	50
moderate health literacy (17-22)	2

### Instrument

**Figure 1:**
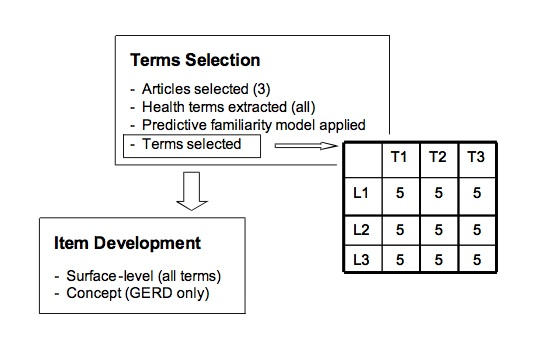
Survey development process (T = topic; L = predicted familiarity level)

A survey for assessing CHV surface-level (45 items) and concept-level (15 items) familiarity was developed, piloted tested, and implemented as described below. The process of instrument development consisted of two stages: (1) selecting health terms for inclusion in the test and (2) developing multiple-choice items for each term (Figure 1).

Candidate CHV terms were selected from consumer health texts for three frequently visited MedlinePlus health topics: hypertension, back pain, and gastroesophageal reflux disease (GERD). One representative article on each selected topic was chosen from among consumer health sites listed by MedlinePlus. A final-year medical student manually extracted all health-related terms from each article. Next, all extracted terms were submitted to the predictive familiarity model [9] and assigned to the categories of “likely,” “somewhat likely,” or “unlikely” to be familiar. Finally, five terms from each predicted familiarity likelihood level were randomly selected from each of the three articles (Multimedia Appendix).

The next stage of instrument construction involved developing multiple-choice test items assessing the two types of familiarity, operationally defined as the following:

1. Surface-level familiarity: ability to match written health terms with basic relevant associated terms at the super-category, location, or function level (eg, “biopsy” is a “test”)

2. Concept-level familiarity: ability to associate written terms with brief phrases describing the meaning or “gists” (eg, “biopsy” means “removing a sample of tissue”)

**Figure 2:**
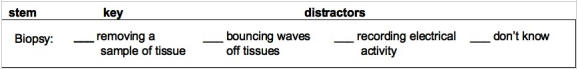
Sample CHV instrument surface-level familiarity item

**Figure 3:**
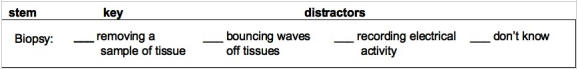
Sample CHV instrument concept-level familiarity item

Surface-level familiarity items (Figure 2) were developed for all selected terms. Concept-level familiarity items (Figure 3) were developed only for the terms extracted from the article on GERD, in order to minimize survey administration time.

The layout of all test items was modeled on the Short Assessment of Health Literacy for Spanish-Speaking Adults (SAHLSA) [11], which in turn is based on the Rapid Estimate of Adult Literacy in Medicine (REALM) health literacy test for English speakers [12]. We chose the SAHLSA model because this validated instrument assesses the ability to associate health terms with other related terms. In contrast, REALM, commonly used in studies with English speakers, only tests the ability to pronounce health terms correctly, which we felt was less appropriate for our purpose of word knowledge assessment. SAHLSA is easy to administer and consists of 50 items, each with a “stem” or target term, a “key” term meaningfully associated with the target term, a “distractor,” and a “don’t know” option. Our only change to this format was adding a second “distractor” to reduce the probability of selecting the “key” term by guessing (see Figure 2). In developing CHV assessment items, we followed the following criteria: (1) the key term and distractors should be of the same difficulty as the target term, (2) distractors should be incorrect but plausible, and (3) the key term and distractors should have the same semantic relationship to the target term (eg, all location or all function). Criteria 2 and 3 were adopted from SAHLSA.

Incorporating the REALM procedure, SAHLSA requires the examinee both to correctly pronounce the target term and to select the key term. However, since our goal was to measure familiarity with written health expressions and concepts explicitly using a self-administered tool (eg, via the Web), the SAHLSA requirement for examinees to pronounce each target expression was dropped. The final test included surface-level familiarity items for all three health topics (questions 1-45) and concept-level familiarity items for GERD terms only (questions 46-60). The entire instrument is available in the Multimedia Appendix.

### Administration, Scoring, and Analysis

Participants first completed the demographics survey, followed by the S-TOFHLA and CHV familiarity survey (surface-level items followed by concept-level familiarity items). For scoring, each correct answer was awarded one point. Surface-level and concept-level familiarity scores were calculated separately. Regression analysis tests on the data were performed at the 0.05 level of significance. Since the study is exploratory in nature, the values between 0.05 and 0.1 are reported for descriptive purposes, as indicating trends for further investigation.

## Results

### Mean Familiarity Scores

Three types of means were computed for each predicted familiarity likelihood level (“likely,” “somewhat likely,” and “unlikely” to be familiar): total surface-level familiarity, GERD surface-level familiarity, and GERD concept-level familiarity (Table 2). Total surface-level familiarity reflects surface-level familiarity with terms on all three topics. Since the test included five terms per topic per level, 15 is the maximum possible surface-level familiarity score for each level. GERD surface-level familiarity indicates surface-level familiarity with GERD terms only, with five the maximum possible score (based on five GERD terms at each level). GERD concept-level familiarity reflects answers to GERD concept questions, with five the maximum possible score for each level.

**Table 2:** Mean surface-level and concept-level familiarity scores

Predicted Familiarity Likelihood	Total Surface-Level Familiarity Mean (SD)	GERD Surface-Level Familiarity Mean (SD)	GERD Concept-Level Familiarity Mean (SD)
Likely	13.80 (1.97)	4.75 (0.81)	3.83 (1.22)
Somewhat likely	12.92 (2.60)	4.54 (1.02)	3.94 (1.04)
Unlikely	9.53 (3.44)	3.42 (1.42)	3.04 (1.31)

Total surface-level familiarity and GERD concept-level familiarity were the dependent variables of hypotheses 1 and 2. GERD surface-level familiarity was used in computing the gap between GERD surface-level and concept-level familiarity, the dependent variable for hypothesis 3.

### Predictors of Total Surface-Level Term Familiarity

Seven independent variables—predicted familiarity likelihood level, gender, English proficiency, highest education level, age, race, and health literacy level (S-TOFHLA scores)—were regressed onto the dependent variable, total surface-level term familiarity score. Linear regression found a statistically significant effect (*P* < .001) of predicted familiarity likelihood level on surface-level term familiarity. Health literacy was another statistically significant predictor of surface-level familiarity (*P* < .001). English proficiency was significant (*P* = .05); education level was not (*P* = .15).

### Predictors of GERD Concept-Level Familiarity

All seven independent variables from the previous regression analysis plus GERD surface-level familiarity were regressed onto GERD concept-level familiarity score. Linear regression found statistically significant effects of predicted familiarity likelihood level (*P* = .009) and GERD surface-level familiarity score (*P* < .001) on GERD concept-level familiarity scores. The effect of health literacy level on GERD concept-level familiarity merits further investigation (*P* = .06).

### Relating GERD Surface-Level and Concept-Level Familiarity Scores

While previous regression analysis indicated that GERD surface-level familiarity score was a significant predictor of GERD concept-level familiarity, the concept-level familiarity consistently lagged behind surface-level familiarity at all three levels (see Table 2). Linear regression analysis of the effect of predicted familiarity likelihood level on the surface-level–concept-level familiarity gap was performed. For the overall model, the gap was statistically significantly different from zero (*P* = .001). In addition, the gap was statistically significantly greater for terms predicted as “likely” then for those “not likely” to be familiar (*P* = .006). The gap for terms predicted as “somewhat likely” versus those predicted “not likely” to be familiar merits further investigation (*P* = .07).

## Discussion

### Implications for the Validity and Usefulness of the CHV Familiarity Model

Although preliminary in nature, this study presents an initial evaluation of the first model for estimating consumer familiarity with health-specific terms. The findings confirmed hypotheses 1 and 3 and partially confirmed hypothesis 2. Confirmation of hypothesis 1 provided initial validity evidence for the CHV familiarity likelihood model [8] by demonstrating a relationship between predicted familiarity and two types of empirically derived consumer familiarity scores. The brief “proof of concept” survey used in this study requires additional research to evaluate the underlying model’s robustness with various target audiences of online consumer health materials: seniors, low-literacy individuals, chronic patients, etc. The approach used in the study provides a methodological framework for such follow-up validation studies. The present study, however, contributes to the field as it suggests that a health corpora frequency-based algorithm presents a feasible and more flexible alternative to general word lists or word length algorithms for estimating the difficulty of consumer health materials. For example, our existing model for predicting term difficulty can be used as a quick screening tool for determining “difficult” terms in consumer health texts and suggesting more consumer-friendly synonyms. Incorporating the model into a formula that produces a single text readability score would potentially automate the complex task of matching consumer health materials to readers (assuming that relevant reader information is available).

### Insights for Improving the Power of CHV Familiarity Prediction

Partial confirmation of hypothesis 2 and confirmation of hypothesis 3 both point to limitations of the model with respect to its ability to identify “consumer-unfriendly” words. Part of the variance in readers’ performance is likely to be related to demographic characteristics, not accounted for in the model. With further research, it is perhaps possible to adjust predicted familiarity likelihood categories for some target populations on the basis of known effects of demographics variables. However, identifying the full range of meaningful demographic variables is not realistic. Moreover, most sites are developed for a broad range of health consumers who represent a diverse range of competencies and experiences. This limitation is not unique to our approach but is true for all attempts to evaluate the difficulty of terms or a text. While individualized prediction of text difficulty on the basis of a model is desirable, it is also much more error prone than population-wide predictions because most predictive models are based on population statistics or empirical expert knowledge. Any prediction is necessarily an approximation, but a high-quality approximation is of considerable value. Presently, our predictive model framework also does not make a theoretical distinction between surface-level familiarity and conceptual understanding and does not make provision for the possible uneven gap between the two. If the uneven gap phenomenon is confirmed, then the “easiness” of terms predicted as highly likely to be familiar may be deceptive. Answering this question requires a strong operational definition of sufficient concept knowledge and a way of assessing it. The present instrument is an exploratory step in the direction of concept knowledge measurement. A satisfactory instrument should reconcile the goals of assessing a complex and multifaceted construct while being relatively quick and easy to administer.

### Limitations of the Study

While most of the study results corresponded to our research hypotheses, the lack of significant effects of most demographic variables, particularly educational level, is surprising and may be due to sampling bias. It is possible that uneven representation obscured any education effects ―41 out of 52 participants had at least some college education. Note that education is a proxy for general literacy, which is only one component of health literacy [10]. Other components, such as health care experience and motivation, may have a much stronger effect on health term familiarity and need to be explored in further research.

### Follow-Up Work

Follow-up work includes validating and possibly adjusting the algorithm for specific populations, evaluating the role of potentially influential demographic variables in designs where these variables are represented across a broad range of values, and developing a formula that would assign a single-value text difficulty on the basis of the present algorithm. The calibration of such formulae in order to estimate the desired scores for various populations would require a set of extensive psychometric studies that are beyond the scope of most informatics research programs. However, developing the algorithm and testing its effectiveness against existing readability formulas are well within the capabilities of consumer health informatics research. It is also essential to develop methods to explore consumer understanding of health concepts in-depth, as the current study only touches the surface of this important topic.

## Multimedia Appendix

60-item questionnaire

## Abbreviations

CHV: consumer health vocabulary
GERD: gastroesophageal reflux disease
SAHLSA: Short Assessment of Health Literacy for Spanish-Speaking Adults
S-TOFHLA: Short Test of Functional Health Literacy in Adults
REALM: Rapid Estimate of Adult Literacy in Medicine
