# Supplementary material for: Assessing Consumer Health Vocabulary Familiarity: An Exploratory Study
Source: J Med Internet Res. 2007 Mar 14;9(1):e5. doi: 10.2196/jmir.9.1.e5 (PMC1874513; doi:10.2196/jmir.9.1.e5)
Supplement: Supplementary file 1 [file jmir_v9i1e5_app1.pdf]

For each item below, check a single word or phrase that is most closely related to the bolded word. If none seem related, check "don't know."

1. Acute
___ short and severe
___ gradual
___ painless
___ don't know

2. Artery
___ nerve
___ muscle
___ blood vessel
___ don't know

3. Chronic
___ immediate
___ long-term
___ burning
___ don't know

4. Diabetes
___ disease
___ test
___ procedure
___ don't know

5. Hormone
___ herbal pill
___ vitamin
___ chemical
___ don't know

6. Amputation
___ exercise
___ injection
___ surgery
___ don't know

7. Pulse
___ heart beat
___ breathing
___ temperature
___ don't know

8. Renal
___ heart
___ kidney
___ lung
___ don't know

9. Spleen
___ organ
___ fluid
___ powerhouse
___ don't know

10. Water Pill
___ birth control
___ placebo (fake pill)
___ medicine
___ don't know

11. Aneurysm
___ urinary tract
___ bones
___ blood vessels
___ don't know

12. Ocular
___ eye
___ ear
___ nerves
___ don't know

13. Diastolic
___ blood pressure
___ temperature
___ breathing
___ don't know

14. Aorta
___ organ
___ bones
___ blood vessels
___ don't know

15. Vasodilator
___ medication

___ device
___ test
___ don't know

16. Cancer
___ broken bone
___ tumor
___ allergy
___ don't know

17. Asthma
___ lungs
___ heart
___ kidneys
___ don't know

18. Muscle
___ taste
___ hearing
___ movement
___ don't know

19. Hospital
___ treatment
___ investment
___ entertainment
___ don't know

20. Surgery
___ sound waves
___ knife
___ camera
___ don't know

21. Intestines
___ chest
___ stomach
___ head
___ don't know

22. Respiratory
___ heat
___ ears
___ lungs
___don't know

23. Ulcer
___ digestive problem
___ breathing problem
___ movement disorder
___ don't know

24. Biopsy
___ treatment
___ test
___ nutrition program
___ don't know

25. Acid reflux
___ stomach
___ eyes
___ urinary tract
___ don't know

26. Pulmonary fibrosis
___ breathing test
___ lung disease
___ bone disease
___ don't know

27. Antacids
___ medicine
___ mouth rinse
___ food supplement
___ don't know

28. Sphincter
___ blood vessels
___ nerves
___ muscles
___ don't know

29. Forceps
___ instrument/device
___ disease
___ medicine (pill)

___ don't know

30. Hiatal hernia
___ intestines problem
___ stomach problem
___ liver problem
___ don't know

31. Prescription drugs
___ food
___ medicine
___ cleaning supplies
___ don't know

32. Lower back
___ body part
___ instrument
___ medical chart
___ don't know

33. Exercise
___ medicine
___ surgery
___ activity
___ don't know

34. Arthritis
___ instrument
___ disease
___ liquid
___ don't know

35. Doctor
___ construction
___ treatment
___ accounting
___ don't know

36. Acupuncture
___ surgery
___ test
___ alternative medicine
___ don't know

37. Flexibility
___ muscles and joints
___ lungs
___ skin
___ don't know

38. Osteoporosis
___ medicine
___ procedure
___ disease
___ don't know

39. Inflammation
___ pain and swelling
___ numbness
___ heavy bleeding
___ don't know

40. Recurrence
___ treatment
___ problem
___ insurance
___ don't know

41. Lumbar
___ lower back
___ shoulders
___ neck
___ don't know

42. Erythrocyte
___ urine
___ blood
___ sweat
___ don't know

43. Fibromyalgia
___ treatment
___ test
___ disease
___ don't know

44. Scoliosis
___ spine
___ throat
___ heart
___ don't know

45. Fascia
___ organ
___ tissue
___ bodily fluid
___ don't know

46. Cancer
___ infection of cells
___ spread of abnormal cells
___ low white blood cell count
___ don't know

47. Asthma
___ inflammation of airways
___ hardening of lungs
___ lung infection
___ don't know

48. Muscle
___ tissue that connects organs
___ tissue that covers organs
___ tissue that can contract
___ don't know

49. Hospital
___ provides medical treatment
___ sells health food
___ educates healthcare investors
___ don't know

50. Surgery
___ getting a blood sample
___ removing or repairing a body part
___ bouncing sound waves off an organ
___ don't know

51. Intestines
___ move food from mouth to stomach
___ digest food
___ remove poisons from blood
___ don't know

52. Respiratory
___ breathing in oxygen
___ breathing in carbon dioxide
___ regulating body temperature
___ don't know

53. Ulcer
___ open sore
___ upset stomach
___ nervous spasm
___ don't know

54. Biopsy
___ bouncing waves off tissues
___ recording electrical activity
___ removing a sample of tissue
___ don't know

55. Acid reflux
___ swallowing problem
___ death of tissue
___ backflow from stomach
___ don't know

56. Pulmonary fibrosis
___ scarring of lungs
___ mucus in lungs
___ fluid in lungs
___ don't know

57. Antacids
___ reduce fever
___ relieve heartburn
___ decrease appetite
___ don't know

58. Sphincter

___ a ring of muscles that opens and closes
___ muscles that help the lungs fill with air
___ a muscle that controls the tongue
___ don't know

59. Forceps
___ measuring blood-pressure
___ numbing a body part
___ holding or removing something
___ don't know

60. Hiatal hernia
___ stomach bulges up into the chest
___ bulging intestines
___ inflammation of the stomach
___ don't know
